# Untargeted Sweat Metabolomics and Targeted Plasma Amino Acid Profiling Reveal Dynamic Metabolic Remodeling During Conditioning in *Yili* Horses

**DOI:** 10.3390/biology15131033

**Published:** 2026-06-28

**Authors:** Yuheng Xue, Penghui Luo, Zhehong Shen, Chen Meng, Xinkui Yao, Jun Meng, Wanlu Ren, Tongliang Wang, Yaqi Zeng

**Affiliations:** 1 College of Animal Science, Xinjiang Agricultural University, Urumqi 830052, China; xyh9523@126.com (Y.X.); 18858657690@163.com (Z.S.); chenmeng0330@126.com (C.M.); yaoxinkui@xjau.edu.cn (X.Y.); junm86@xjau.edu.cn (J.M.); renwanlu@xjau.edu.cn (W.R.); wtl13639911402@163.com (T.W.); 2 Xinjiang Key Laboratory of Horse Breeding and Exercise Physiology, Urumqi 830052, China; 3 Horse Industry Research Institute, Xinjiang Agricultural University, Urumqi 830052, China; 4 Xinjiang Uygur Autonomous Region Animal Husbandry General Station, Urumqi 830052, China; xmzztxzx1@163.com

**Keywords:** *Yili* horse, conditioning training, sweat morphology, plasma amino acid, metabolomics, training adaptation

## Abstract

Training induces sweat morphology changes in horses from muddy sweat (MS) to foamy sweat (FS) and clear sweat (CS), reflecting physiological adaptation. However, the metabolic mechanisms linking sweat phenotypes to systemic amino acid dynamics remain unclear. This study integrated sweat untargeted metabolomics and plasma amino acid targeted metabolomics to reveal coordinated metabolic remodeling. Six 2-year-old *Yili* horse stallions underwent 10-week training. Plasma and sweat were sampled pre- and post-race at each stage. LC-MS/MS and UHPLC-MS/MS were used for sweat metabolome and plasma amino acid analysis, followed by multivariate statistics, KEGG enrichment, and correlation network (CNet) analysis. Differential sweat metabolites decreased across stages (45, 127, and 38 for MS vs. FS, MS vs. CS, and FS vs. CS). Pre-race BCAA concentrations were higher in MS than in FS and CS, while glycine was lower. Post-race valine, histidine, and aspartate were elevated only in MS. Pre-race plasma amino acids positively correlated with sweat lipids and organic acids, shifting to negative post-race. ABC transporters, mTOR signaling, and BCAA metabolic pathways were key co-regulators. The MS-to-CS transition reflects metabolic remodeling from acute stress to homeostatic adaptation. Plasma BCAAs and sweat cortisol are potential biomarkers, with ABC transporters and mTOR pathways mediating sweat-plasma metabolic coordination.

## Simple Summary

Horses sweat to cool down during exercise, and the appearance of their sweat changes as they get fitter. In young *Yili* horses undergoing training, sweat initially appears as a thick, muddy form; then, it becomes foamy, and finally turns into a thin clear liquid. We wanted to understand what these sweat changes tell us about how the horse’s body adjusts to exercise. We collected sweat and blood samples from six horses at each sweat stage and used advanced laboratory techniques to identify the molecules present. We found that horses with muddy sweat had higher blood concentrations of branched-chain amino acids, which are protein building blocks that serve as muscle fuels, and lower concentrations of another protein building block called glycine, compared to horses with foamy or clear sweat. After exercise, only the muddy-sweat horses showed continued blood chemistry changes. We also identified biological systems that link sweat chemistry with blood nutrients, including ABC transporters, which are proteins that help move amino acids and other small molecules from the blood into the sweat, and the mTOR signaling pathway, which controls muscle growth. A stress hormone called cortisol in sweat could serve as a simple way to check how stressed a horse is from training. These findings show that horse trainers can simply observe sweat appearance to judge training progress, avoiding blood sampling and reducing stress on the horses, while improving animal welfare.

## 1. Introduction

The *Yili* horse is a renowned Chinese indigenous breed native to the Ili Kazakh Autonomous Prefecture in Xinjiang, China. Historically developed as a dual-purpose breed for riding and draft work, *Yili* horses have gained increasing importance in modern Chinese equine sports, particularly racing and endurance events. Their adaptation to the high-altitude alpine environment of the Zhaosu region and their well-documented sweating physiology make them an excellent model for studying exercise-induced metabolic remodeling.

Horses are among the few mammalian species that rely primarily on sweating for thermoregulation during exercise. During conditioning, as exercise intensity and training phases progress, horses undergo a series of adaptive changes involving multiple systems and organs [1,2]. The morphological change of sweat is a common physiological phenomenon during the training of sport horses; at the initial stage of training, horse sweat is mostly muddy sweat, which gradually transitions to foamy sweat and finally forms clear water sweat as training advances [3,4]. These morphological transitions not only reflect the horse’s adaptation to training load but may also be closely related to the excretion of metabolic products [5,6,7]. However, whether these transitions merely reflect changes in the surfactant protein latherin concentration or signal broader systemic metabolic reprogramming remains controversial.

Amino acids serve as the basic constituents of proteins and play crucial physiological roles during exercise, participating not only in protein synthesis but also serving as energy substrates and mediating metabolic regulation under exercise stress [8,9]. Notably, branched-chain amino acids (BCAAs), including leucine, isoleucine, and valine, function as both direct energy sources and signaling molecules that regulate muscle protein synthesis through the mammalian target of rapamycin (mTOR) pathway. Changes in plasma amino acid concentrations during conditioning can reflect the metabolic response to exercise load [9,10]. The amino acid compositions of horse sweat and plasma differ [11]. Following the standardized 2000 m test race, serum concentrations of branched-chain amino acids and alanine changed markedly, indicating that amino acid metabolism constitutes a central node of the equine exercise response [12]. Previous metabolomics studies in horses have primarily focused on Thoroughbreds and Standardbreds, investigating exercise-induced changes in plasma metabolites and energy metabolism [8,10]. For example, plasma amino acid profiles have been characterized in endurance and racing horses, revealing alterations in BCAA and aromatic amino acid concentrations following intense exercise [9,12]. However, comparable investigations in Chinese indigenous breeds such as the *Yili* horse are lacking. Furthermore, the integration of sweat metabolomics with circulatory metabolite profiling has not been previously reported in any equine breed, representing a significant gap in our understanding of the circulatory-excretory metabolic axis during conditioning. While blood and sweat metabolomes have been extensively characterized as separate compartments, the coordinated metabolic interplay between systemic circulation and exocrine secretion during progressive conditioning remains largely unexplored. This analytical disjunction carries practical implications because sweat provides a non-invasive window into the coordinated metabolic responses of systemic circulation and eccrine secretion during exercise, affording a means to monitor training adaptation without blood sampling.

This study used *Yili* horses as a model, integrating untargeted sweat metabolomics and targeted plasma amino acid metabolomics to (i) characterize sweat metabolic signatures associated with each sweat phenotype, (ii) identify plasma amino acid patterns distinguishing different training adaptation stages, and (iii) elucidate the key pathways connecting circulatory and exocrine metabolic responses. By elucidating the metabolic connections between sweat phenotypes and circulatory amino acid dynamics, this study aims to establish a non-invasive framework for monitoring training adaptation and optimizing conditioning protocols in sport horses.

## 2. Materials and Methods

### 2.1. Animals and Training Protocol

This experiment was conducted from July to October 2023 at Zhaosu Stud Farm, Zhaosu County, Xinjiang, China. All horses, trainers, jockeys, and the test track were provided by the Zhaosu Stud Farm. The test track was an oval sand track with a width of 14 m and a circumference of 1000 m. The detailed training protocol is provided in Supplementary Table S1.

Six 2-year-old *Yili* horse stallions were selected from the Zhaosu Stud Farm, Ili Kazakh Autonomous Prefecture, Xinjiang Uygur Autonomous Region. All horses had similar body measurements, were in good health, and were maintained under identical environmental conditions. During the experiment, horses were housed individually and fed 1.5 kg of green hay at 07:00, 12:30, 16:00, 19:30, and 24:00, and 1 kg of concentrate at 07:30 and 20:30. Water was provided ad libitum throughout the day. Stables were disinfected and manure was cleaned daily.

All six horses were washed with Safeguard body wash (Procter & Gamble, Cincinnati, OH, USA) to ensure complete cleanliness before commencing a 10-week conditioning program. A standardized 2000 m test race was organized on the third day following the complete morphological change of sweat in each horse. The test race was conducted at a track temperature of 20–23 °C and a relative humidity of 65–75% RH.

Housing and husbandry. Horses were housed at the Zhaosu Stud Farm in individual box stalls (approximately 3.5 × 3.5 m) with straw bedding that was cleaned and replaced every two days. Horses were turned out to outdoor paddocks for approximately 4 h daily during daylight hours. The facility maintained natural ventilation and a natural light/dark cycle corresponding to the local photoperiod (July to October, approximately 14–11 h light/10–13 h dark). Ambient temperature during the experimental period ranged from approximately 8 to 24 °C. Horses were fed a standard racing horse diet consisting of alfalfa hay and mountain grass hay provided ad libitum, supplemented with a commercial concentrate feed provided at approximately 3.0 kg per horse per day, divided into three equal portions fed at 07:00, 13:00, and 18:00. Fresh water was available ad libitum from automatic waterers.

All horses initially presented muddy sweat (MS) at the beginning of the conditioning program. The transition from MS to foamy sweat (FS) typically occurred around week 3–4 of training, and the transition from FS to clear sweat (CS) occurred around week 7–8. Thus, the sequential sweat morphology transition served as a non-invasive indicator of progressive training adaptation.

This study employed a longitudinal within-subject design where each horse served as its own control. The three sweat stages (MS, FS, CS) represent sequential measurements from the same six horses over the 10-week training period, rather than independent groups. This design was chosen to minimize inter-individual variability and to capture the temporal dynamics of metabolic adaptation within individual animals.

### 2.2. Sample Collection

The main equipment used included the following: Polar Grit X heart rate monitor(Polar Electro Oy, Kempele, Finland), sweat collection patch (comprising sterile PU film tape (3M Health Care, St. Paul, MN, USA), absorbent cotton (Winner Medical, Shenzhen, China), sterile gauze (Winner Medical, Shenzhen, China)), 5 mL blood collection tubes with blood collection needle (BD Vacutainer, Becton Dickinson, Franklin Lakes, NJ, USA), 20 mL centrifuge tubes (Eppendorf, Hamburg, Germany), 1000 µL pipette (Eppendorf, Hamburg, Germany), high-definition camera (Canon EOS 500D, Canon Inc., Tokyo, Japan), stopwatch (Casio, Tokyo, Japan), Jingbo 80-2 desktop electric centrifuge (Jingbo Scientific Instruments, Taizhou, China), BA200 automatic biochemical analyzer (Biosystems S.A., Barcelona, Spain), Huaweidelang DR-200BS enzyme-linked analyzer (Wuxi Huawei Delang Instrument Co., Ltd., Wuxi, China), 5 mL cryogenic storage tubes (Corning, NY, USA), thermal container (Haier, Qingdao, China), and liquid nitrogen tank (Chengdu Jinfeng Instrument Co., Ltd., Chengdu, China).

Plasma collection: On the test race day, 10 mL of venous blood was collected using a blood collection needle (BD Vacutainer, Becton Dickinson, Franklin Lakes, NJ, USA) and a heparin sodium anticoagulant tube (BD Vacutainer, Becton Dickinson, Franklin Lakes, NJ, USA) at 3 h after morning feeding (pre-race, resting state) and immediately after the race. Plasma was obtained by centrifugation at 3000 r/min for 15 min, transferred to cryogenic storage tubes (Corning, NY, USA), flash-frozen in liquid nitrogen for 15 min, and subsequently stored at −80 °C.

Blood samples were collected within 30 min after the horses completed the 2000 m test race, without a standardized cool-down period. Prior to sweat collection, the skin surface was thoroughly washed with Safeguard body wash (Procter & Gamble, Cincinnati, OH, USA) to remove dirt and contaminants, followed by repeated rinsing with distilled water to ensure complete removal of residual cleansing agents.

**Figure 1:**
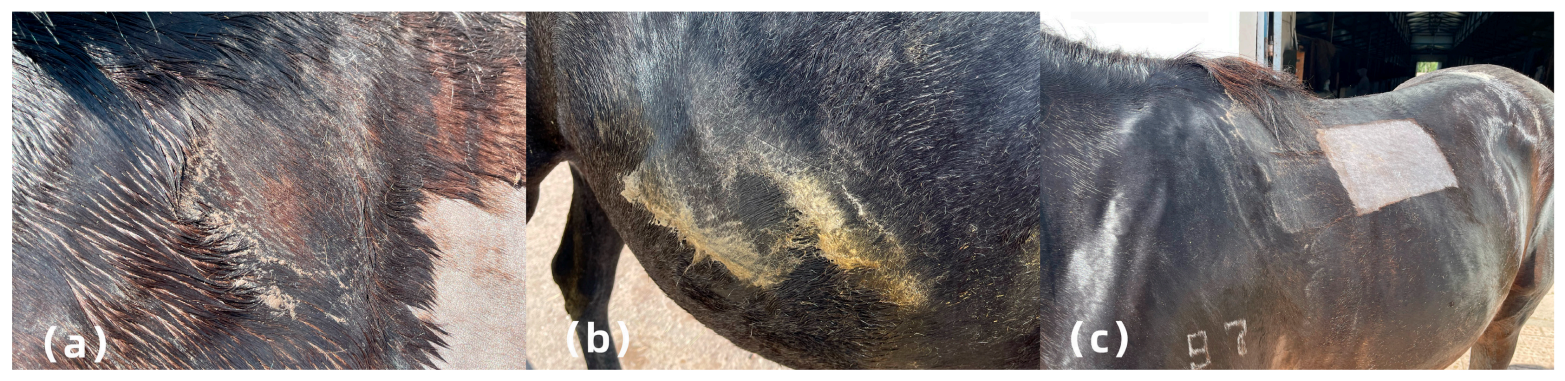
Sweat morphologies of *Yili* horses during conditioning. (**a**) Muddy sweat (MS): turbid, viscous sweat mixed with dirt and debris, observed at the initial stage of conditioning (~0 days). (**b**) Foamy sweat (FS): white, foamy sweat caused by latherin protein and lipid interaction, appearing around day 20 of conditioning. (**c**) Clear sweat (CS): thin, transparent sweat with high evaporative efficiency, appearing around day 55 of conditioning.

Sweat morphology observation: At the initial stage of conditioning, the horses predominantly exhibited muddy sweat (MS, group A), which was turbid in color and mixed with dirt, with an astringent odor. Approximately 20 days into training, the horses began to produce foamy sweat (FS, group B), which appeared white and foamy, with an odor resembling alkaline water. Around day 55, the horses produced clear sweat (CS, group C), which was relatively transparent, with a similar alkaline odor (Figure 1; Table 1).

**Figure 2:**
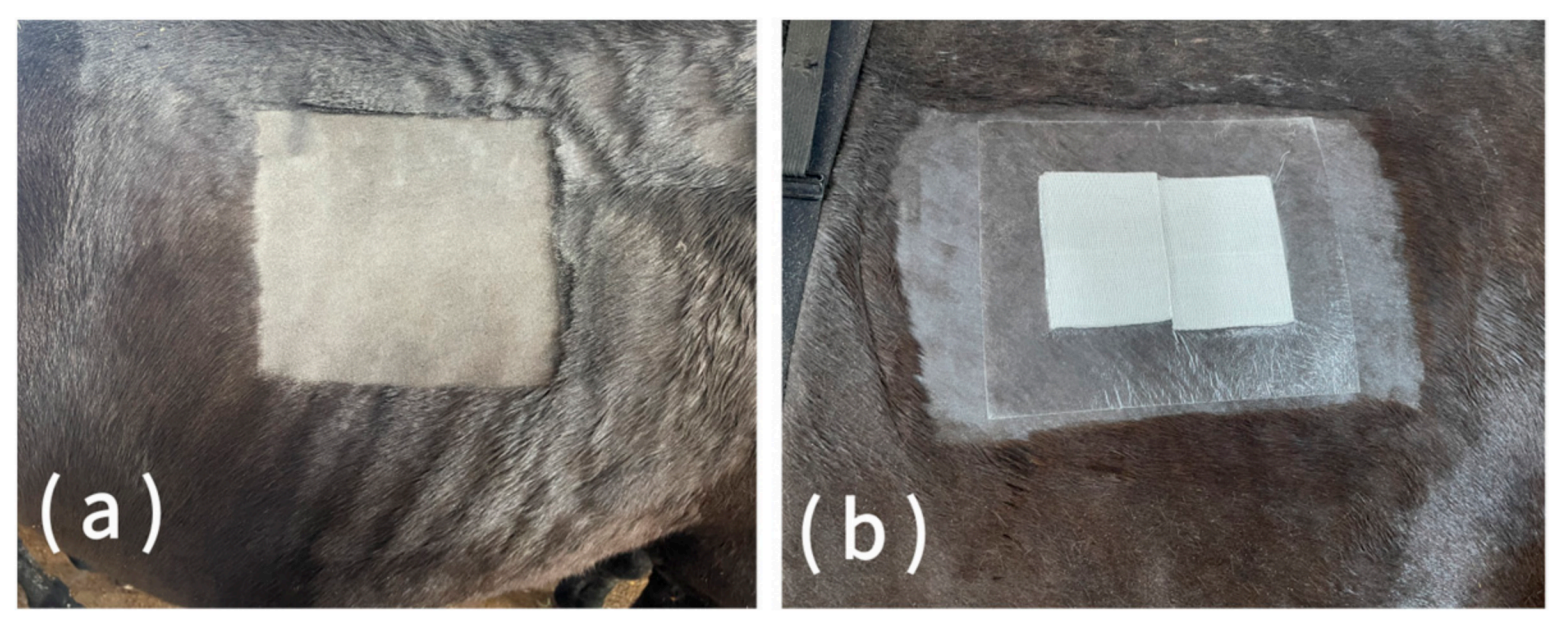
Sweat collection area and patch application. (**a**) The hair was shaved from the 9th to 16th rib region on both sides of the back to expose the skin. (**b**) A sweat collection patch was applied to the exposed skin.

Sweat collection: One day prior to the test race, hair was shaved from both sides of the back at the 9th to 16th rib region to expose the skin (Figure 2a). Before the race, the exposed skin was cleaned with 75% alcohol and distilled water (500 mL per wash) for three times. After air-drying, a sweat collection patch was applied to the exposed skin (Figure 2b). The sweat collection area corresponded to the absorbent pad area of the patch (0.0192 m^2^). Immediately after the race, the absorbent pad was removed and sweat was collected by centrifugal filtration. A high-definition camera was used to photograph and document the sweat morphology of each horse.

### 2.3. Metabolomics Sequencing and Analysis

Sweat untargeted metabolomics: Sweat samples were analyzed by liquid chromatography tandem mass spectrometry (LC-MS/MS; Thermo Fisher Scientific, Waltham, MA, USA). Metabolites were extracted using organic solvent precipitation, followed by non-targeted metabolomic detection. Chromatographic separation was performed on a Hypersil Gold C18 column (Thermo Fisher Scientific, Waltham, MA, USA) at 40 °C with a flow rate of 0.2 mL/min. For positive ion mode, mobile phase A was 0.1% formic acid (Thermo Fisher Scientific, Waltham, MA, USA); for negative ion mode, mobile phase A was 5 mmol/L ammonium acetate (Thermo Fisher Scientific, Waltham, MA, USA) (pH 9.0). Mobile phase B was methanol (Thermo Fisher Scientific, Waltham, MA, USA) in both modes. The elution gradient was as follows: 0–3 min, 2% B; 3–10 min, 100% B; 10.1–12 min, 2% B. The mass spectrometer was operated in the following conditions: scan range m/z 100–1500; electrospray ionization source with a spray voltage of 3.5 kV; sheath gas flow rate of 35 psi; auxiliary gas flow rate of 10 L/min; ion transfer tube temperature of 320 °C; and auxiliary gas heater temperature of 350 °C. A 53% methanol aqueous solution (prepared in-house) was used as the blank sample.

Plasma targeted amino acid metabolomics: An ultra-high performance liquid chromatography coupled to tandem mass spectrometry (UHPLC-MS/MS) system (ExionLC™ AD UHPLC-QTRAP 6500+, AB SCIEX Corp., Boston, MA, USA) was used to quantitate amino acids (provided by Novogene Co., Ltd., Beijing, China). Separation was performed on an ACQUITY UPLC BEH Amide column (Waters Corporation, Milford, MA, USA) (2.1 × 100 mm, 1.7 µm) maintained at 50 °C. The mobile phase consisted of 0.1% formic acid in 5 mM ammonium acetate (solvent A; Thermo Fisher Scientific, Waltham, MA, USA) and 0.1% formic acid in acetonitrile (solvent B; Thermo Fisher Scientific, Waltham, MA, USA), delivered at a flow rate of 0.30 mL/min. The solvent gradient was: initial 80% B, 0.5 min; 80–70% B, 2 min; 70–45% B, 4 min; 45–80% B, 6.01 min; 80% B, 9 min. The mass spectrometer was operated in positive multiple reaction monitoring (MRM) mode with the following parameters: IonSpray Voltage 5500 V, Curtain Gas 35 psi, Ion Source Temp 550 °C, Ion Source Gas 1 and 2 at 50 and 60 psi, respectively.

Multivariate statistical analysis: Principal component analysis (PCA), partial least squares discriminant analysis (PLS-DA), and orthogonal partial least squares discriminant analysis (OPLS-DA) were employed to explore metabolic profiles. Differential metabolite identification: Differential metabolites were identified based on variable importance in projection (VIP) score, fold change (FC), and *t*-test *p*-value. The criteria for significant differential metabolites were VIP > 1, FC > 1.2 or FC < 0.833, and *p* < 0.05. Volcano plots were used to visualize differential metabolite expression patterns. Pathway enrichment analysis: The Kyoto Encyclopedia of Genes and Genomes (KEGG) database was used for pathway enrichment analysis of differential metabolites. Sankey bubble plots were generated to display enriched metabolic pathways and the differential metabolites enriched within them, revealing the metabolic effects of training. Correlation analysis: Chord diagrams were constructed to reveal correlations between sweat and plasma metabolites, providing an intuitive visualization of the relationships and conversions among different metabolites. Co-enrichment pathway correlation network (CNet) diagrams were further constructed to explore the connections between sweat-plasma metabolically co-enriched pathways.

All statistical analyses were performed using R software (version 4.4.1, R Foundation for Statistical Computing, Vienna, Austria), with the ggplot2 (version 3.5.1) and ropls (version 1.40.0) packages.

### 2.4. Ethics Statement

All animal experiments were conducted in accordance with the Guidelines for the Care and Use of Experimental Animals established by the Ministry of Science and Technology of the People’s Republic of China. The study protocol was reviewed and approved by the Animal Welfare and Ethics Committee of Xinjiang Agricultural University (approval no. 2023037, dated 16 July 2023).

## 3. Results

### 3.1. Sweat Untargeted Metabolomic Analysis

**Figure 3:**
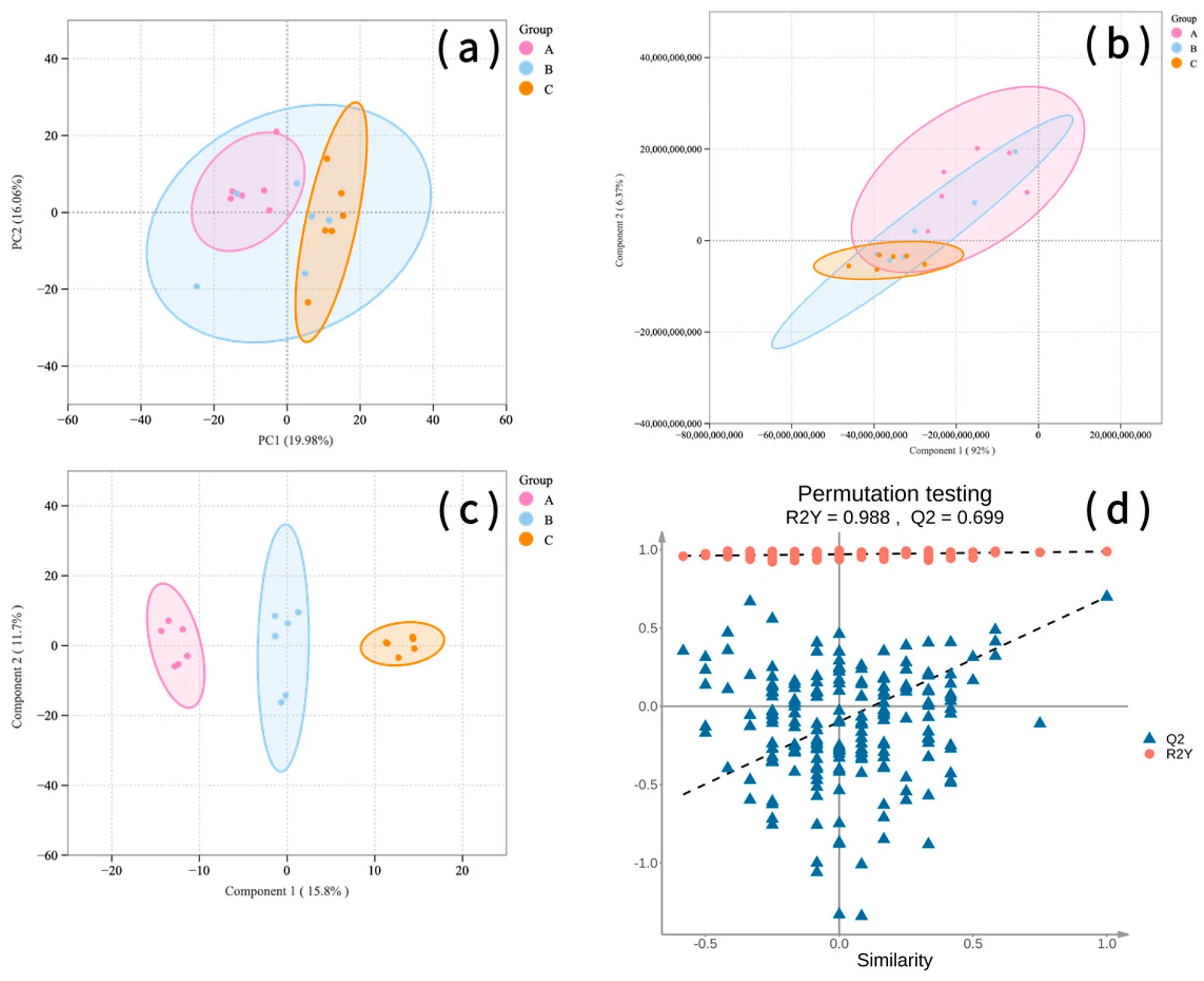
Sweat untargeted metabolomic analysis across three sweat stages. (**a**) PCA score plot; (**b**) PLS−DA score plot; (**c**) OPLS−DA score plot; (**d**) OPLS−DA permutation test (R^2^Y = 0.988, Q^2^ = 0.699). In all panels, muddy sweat (MS) is represented in pink, foamy sweat (FS) in blue, and clear sweat (CS) in orange.

**Figure 4:**
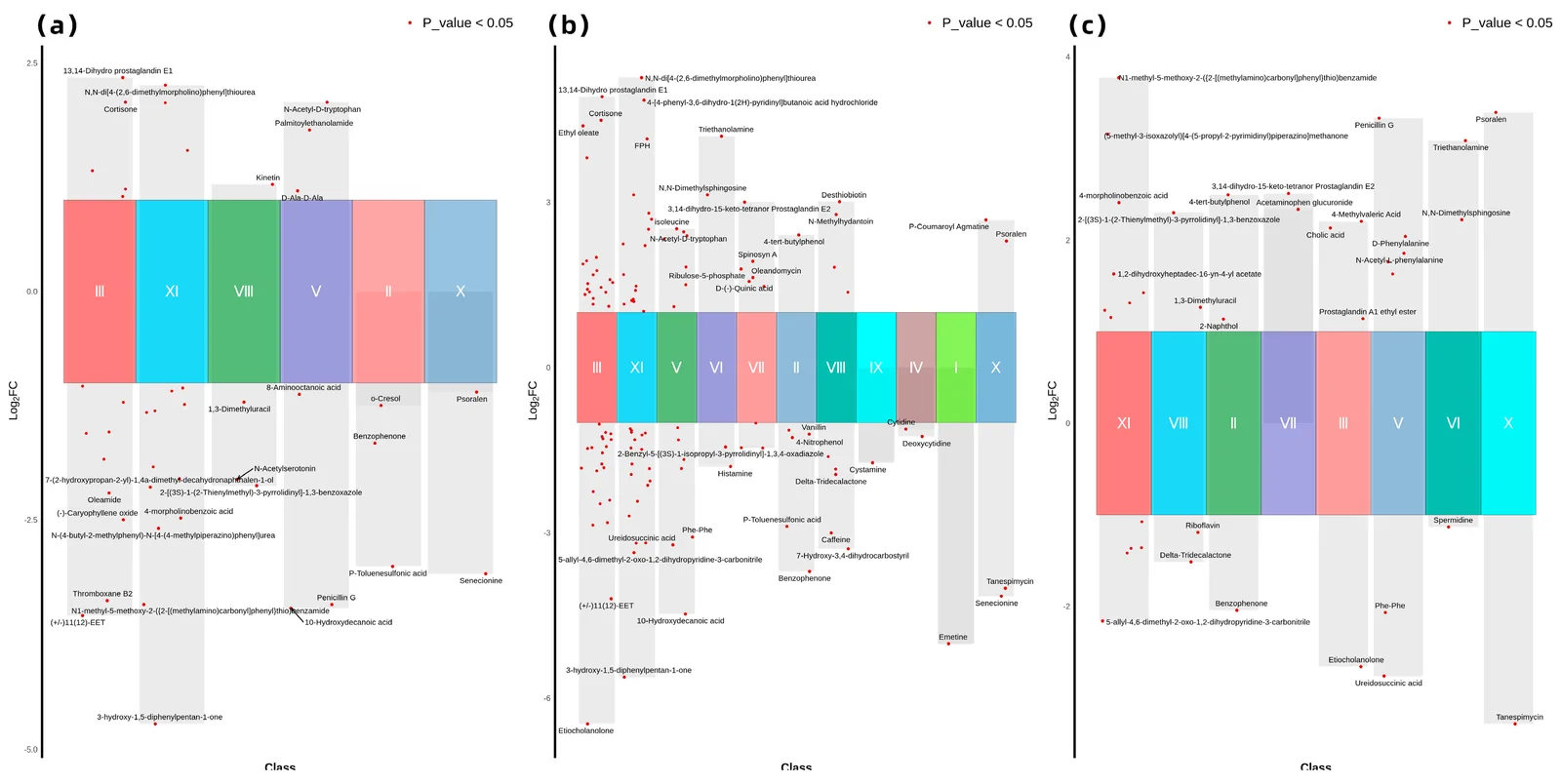
Sweat untargeted metabolomic analysis across three sweat stages. (**a**) A vs. B differential metabolite classification volcano plot; (**b**) A vs. C differential metabolite classification volcano plot; (**c**) B vs. C differential metabolite classification volcano plot. Superclass abbreviations: I, Alkaloids and derivatives; II, Benzenoids; III, Lipids and lipid-like molecules; IV, Nucleosides, nucleotides, and analogues; V, Organic acids and derivatives; VI, Organic nitrogen compounds; VII, Organic oxygen compounds; VIII, Organoheterocyclic compounds; IX, Organosulfur compounds; X, Phenylpropanoids and polyketides; XI, Other.

A series of multivariate statistical analyses were employed to assess the overall differences and trends in sweat metabolites across the three sweat stages. Principal component analysis (PCA) revealed that the three groups—muddy sweat (A), foamy sweat (B), and clear sweat (C)—did not achieve complete separation on the score plot, indicating a degree of metabolic overlap among groups; however, a discernible grouping trend was observed, suggesting that sweat metabolite profiles changed progressively with sweat morphology (Figure 3a). To further amplify inter-group differences and identify potential biomarkers, supervised partial least squares discriminant analysis (PLS-DA) was applied. The PLS-DA model markedly improved group separation, with samples from each group forming distinct clusters, indicating that the model effectively captured key metabolite information capable of differentiating sweat morphologies (Figure 3b). We further constructed an orthogonal partial least squares discriminant analysis (OPLS-DA) model, which provided clearer visualization of inter-group differences (Figure 3c). To validate model reliability, a 200-time permutation test was performed. The results showed that all Q^2^ values of the original models exceeded those of the permuted models, with the regression line intercept at Q^2^ = 0.699, robustly demonstrating that the OPLS-DA model was stable and free from overfitting (Figure 3d). The PCA score plots showed partial overlap between groups, which is expected given the progressive and continuous nature of physiological adaptation to training. The sweat morphology transition represents a gradual metabolic remodeling process rather than discrete, abrupt shifts. PLS-DA and OPLS-DA were therefore employed to maximize group discrimination by incorporating class information into the multivariate model. Model validation parameters (R^2^Y and Q^2^ values) indicated acceptable model robustness for interpretation. Based on the OPLS-DA model, combined with VIP > 1 and *p* < 0.05, differential metabolites were screened for each comparison group. In the A vs. B comparison, a total of six categories and 45 significant differential metabolites (*p* < 0.05) were identified (Figure 4a). Among lipids and lipid-like molecules, 1-stearoylglycerol (FC = 2.05) and four other substances were significantly upregulated (*p* < 0.05), whereas thromboxane B2 (FC = 0.10) and oleamide (FC = 0.22) and eight other substances were significantly downregulated. In organic acids and derivatives, D-Ala-D-Ala (FC = 2.15) and two other substances were upregulated, while 8-aminooctanoic acid (FC = 0.46) and two other substances were downregulated.

**Figure 5:**
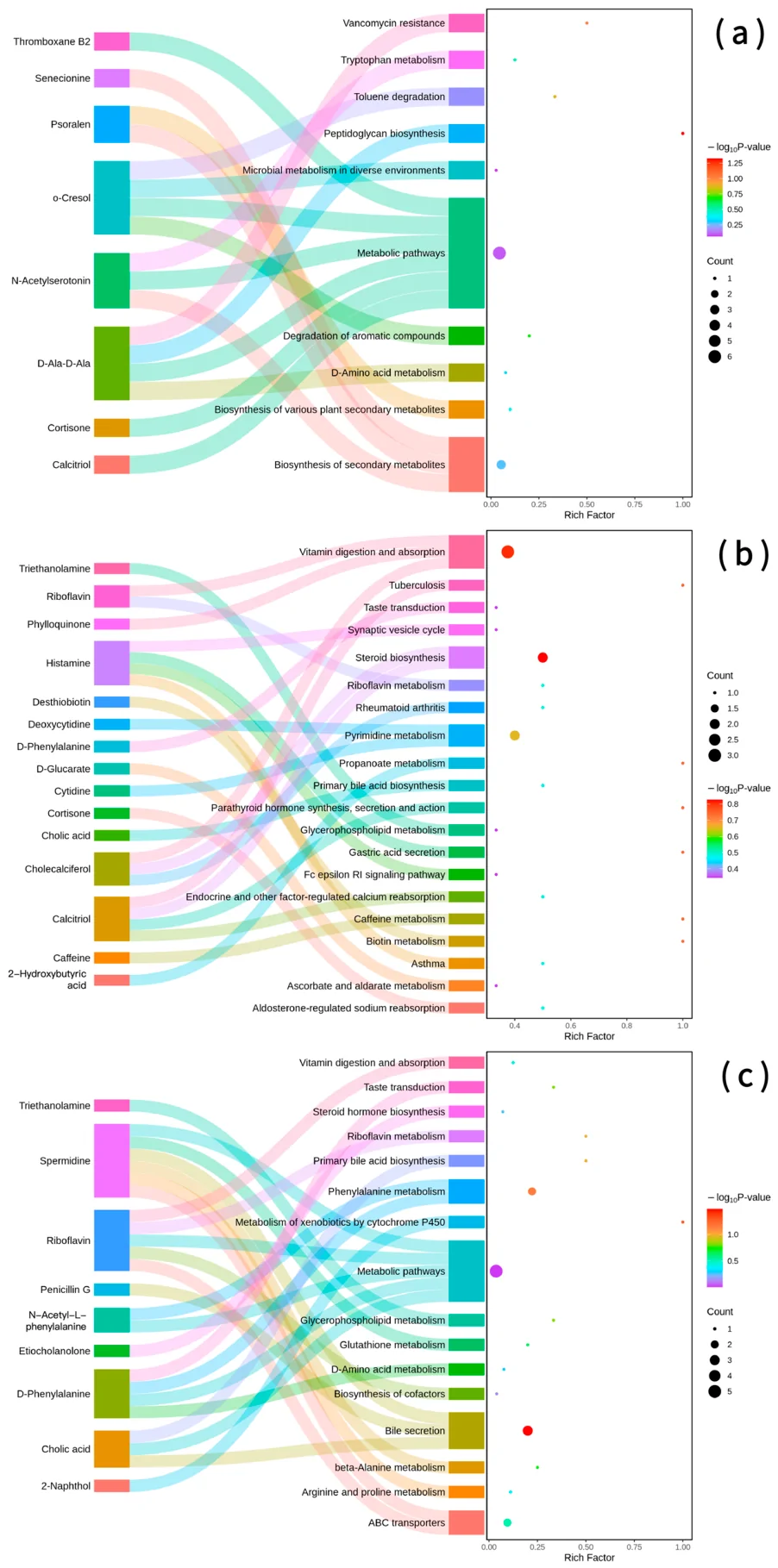
Sweat untargeted metabolomic analysis across three sweat stages. (**a**) A vs. B Sankey bubble plot; (**b**) A vs. C Sankey bubble plot; (**c**) B vs. C Sankey bubble plot.

In the A vs. C comparison, 11 categories and 127 differential metabolites were identified (Figure 4b). Among lipids and lipid-like molecules, cortisone (FC = 22.44) and cholic acid (FC = 13.99) and 18 other substances were upregulated, while adrenic acid (FC = 0.40) and coenzyme Q2 (FC = 0.28) and 19 other substances were significantly downregulated (*p* < 0.05). In organic acids and derivatives, isoleucine (FC = 5.73) and six other substances were significantly upregulated(*p* < 0.05), while ureidosuccinic acid (FC = 0.04) and six other substances were significantly downregulated(*p* < 0.05). In the B vs. C comparison, the number of differential metabolites decreased to 8 categories and 38 (Figure 4c), indicating that the metabolic state of the horses was stabilizing. Sankey bubble plots of enriched pathways for each comparison group are shown in Figure 5a–c. In A vs. B, the main enriched pathways included vancomycin resistance, toluene degradation, peptidoglycan biosynthesis, and tryptophan metabolism, with differential metabolites D-Ala-D-Ala, N-acetylserotonin, and o-cresol being significantly enriched (*p* < 0.01) (Figure 5a). In A vs. C, the main enriched pathways included vitamin digestion and absorption, parathyroid hormone synthesis, and steroid biosynthesis, with riboflavin, phylloquinone, vitamin D3, and calcitriol being significantly enriched (*p* < 0.01) (Figure 5b). In B vs. C, the main enriched pathways included bile secretion, cytochrome P450 metabolism of xenobiotics, and phenylalanine metabolism, with cholic acid, spermidine, 2-naphthol, and riboflavin being significantly enriched (*p* < 0.01) (Figure 5c).

### 3.2. Plasma Targeted Amino Acid Metabolomic Analysis

**Figure 6:**
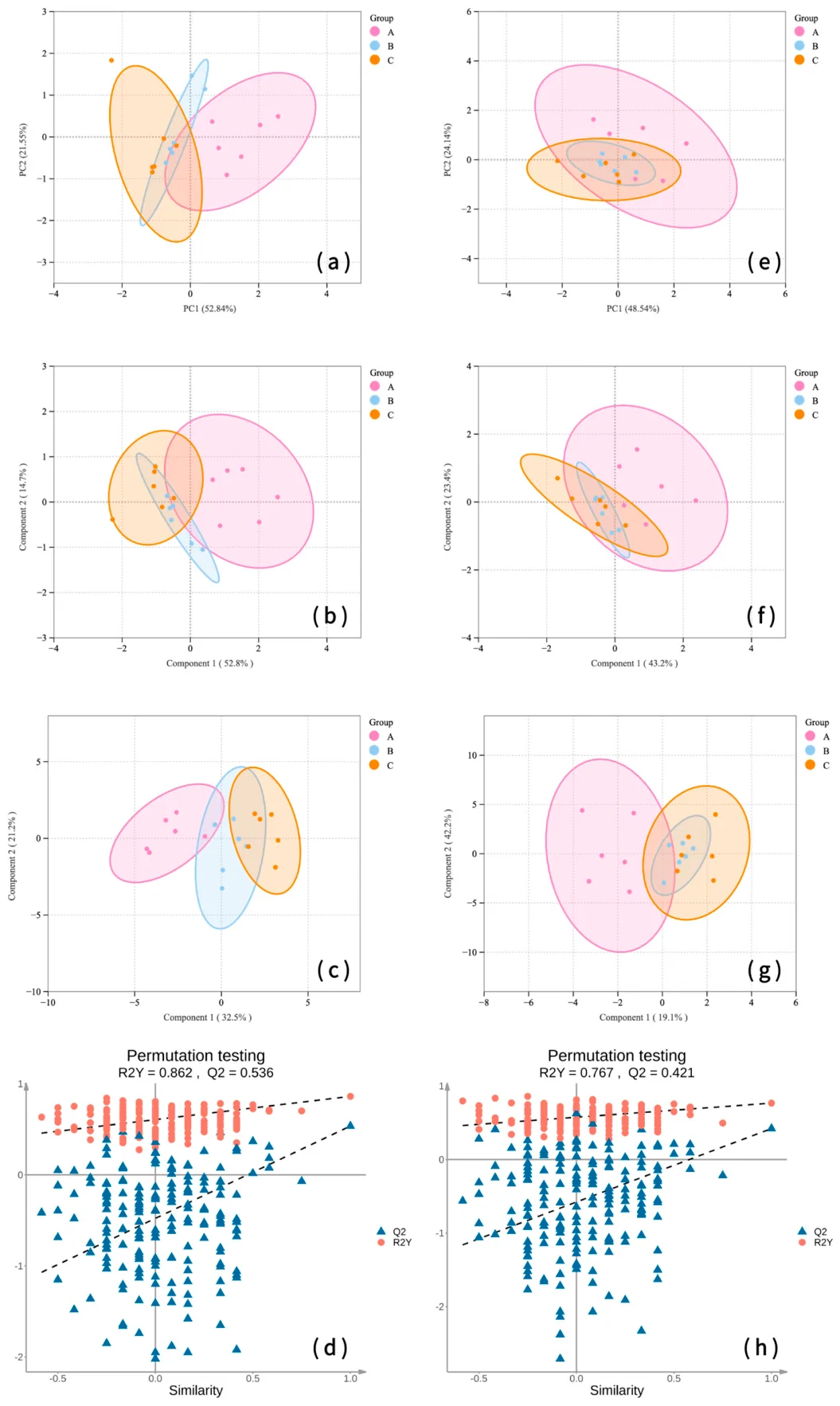
Multivariate statistical analysis of plasma targeted amino acid metabolomics. Left column (**a**–**d**): pre-race (resting state); right column (**e**–**h**): post-race. (**a**,**e**) PCA score plots; (**b**,**f**) PLS-DA score plots; (**c**,**g**) OPLS-DA score plots; (**d**) OPLS-DA permutation test for pre-race comparison (R^2^Y = 0.862, Q^2^ = 0.536); (**h**) OPLS-DA permutation test for post-race comparison (R^2^Y = 0.767, Q^2^ = 0.421). In all panels, muddy sweat (MS) is represented in pink, foamy sweat (FS) in blue, and clear sweat (CS) in orange.

A series of multivariate statistical methods were used to evaluate the overall differences and trends in plasma amino acids before and after the test race. PCA initially revealed a potential clustering trend among samples, but complete separation was not achieved, suggesting that non-supervised models alone were insufficient to capture the subtle metabolic changes induced by sweat morphology differences (Figure 6a,e). PLS-DA improved group separation to some extent, but the effect remained limited, indicating the possible interference of orthogonal variables unrelated to grouping (Figure 6b,f). OPLS-DA models clearly showed separation trends among groups, demonstrating that horses with different sweat morphologies exhibited intrinsic differences in plasma amino acid metabolism (Figure 6c,g). Permutation test results showed Q^2^ values of 0.536 and 0.621 for the two key comparisons, confirming stable and reliable models without overfitting risk (Figure 6d,h). Differential amino acids were screened based on the criteria of VIP > 1, FC > 1.2 or < 0.833, and *p* < 0.05.

**Figure 7:**
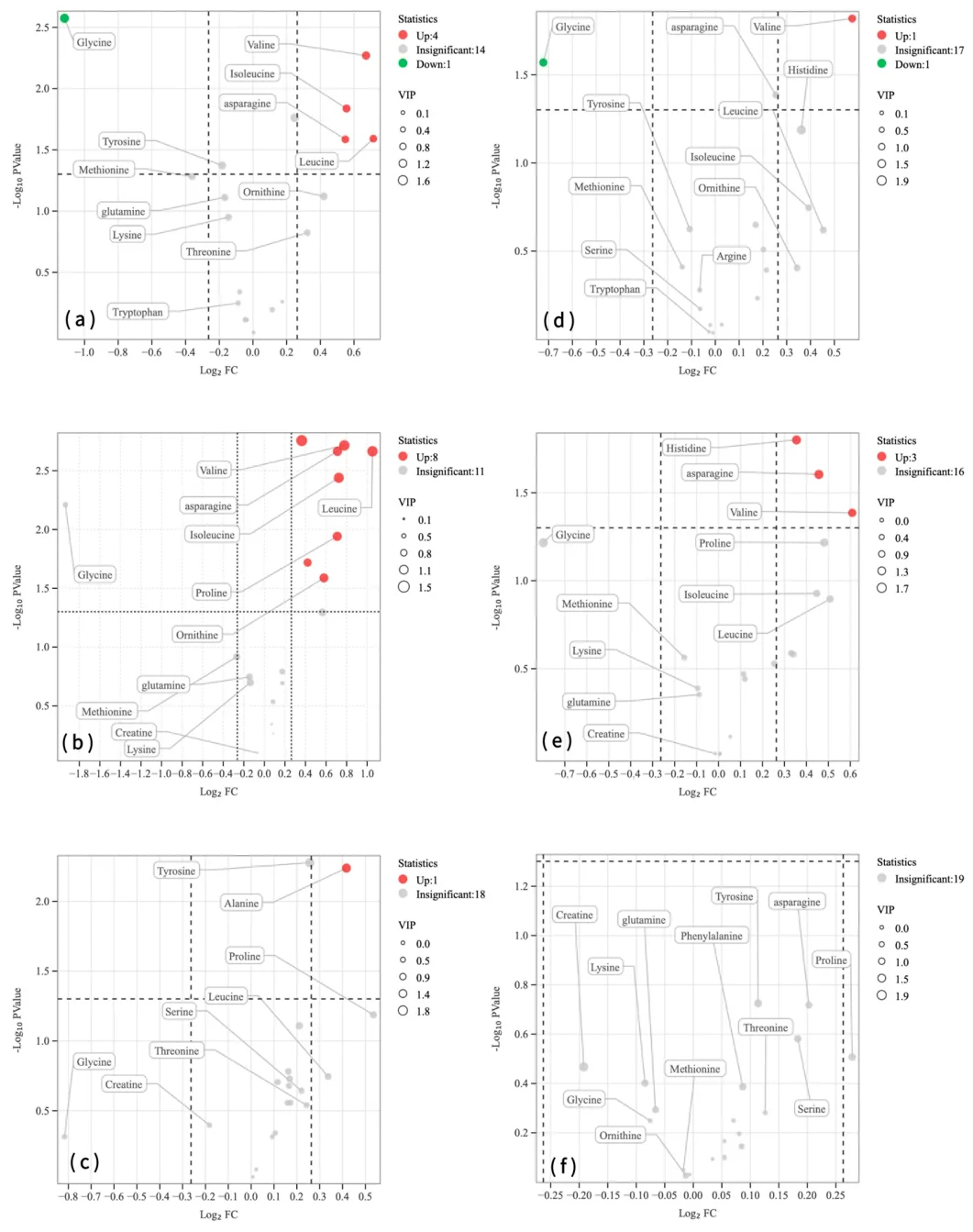
Differential amino acid volcano plots. (**a**) Resting A vs. B; (**b**) Resting A vs. C; (**c**) Resting B vs. C; (**d**) Post−race A vs. B; (**e**) Post−race A vs. C; (**f**) Post−race B vs. C. Red dots indicate significantly upregulated amino acids (VIP > 1, FC > 1.2, *p* < 0.05); green dots indicate significantly downregulated amino acids (VIP > 1, FC < 0.833, *p* < 0.05); gray dots indicate no significant difference. The size of each dot represents the VIP value.

Differential amino acids were identified based on the criteria of VIP > 1, fold change (FC) > 1.2 or < 0.833, and *p* < 0.05. All reported significant differences met this threshold unless otherwise noted. Targeted metabolomics detected a total of 19 amino acids. In the pre-race resting state, the volcano plot for A vs. B showed that valine (Val), leucine (Leu), isoleucine (Ile), and aspartate (Asp) were significantly upregulated, while glycine (Gly) was significantly downregulated (Figure 7a). In A vs. C, 8 amino acids including Val, Leu, and Ile were significantly upregulated, while Gly was downregulated but not significantly (Figure 7b). In B vs. C, only alanine (Ala) was significantly upregulated (Figure 7c). Post-race, A vs. B showed Val significantly upregulated and Gly significantly downregulated (Figure 7d). In A vs. C, Val, histidine (His), and Asp were significantly upregulated (Figure 7e). In B vs. C, no differential amino acids were identified (Figure 7f).

**Figure 8:**
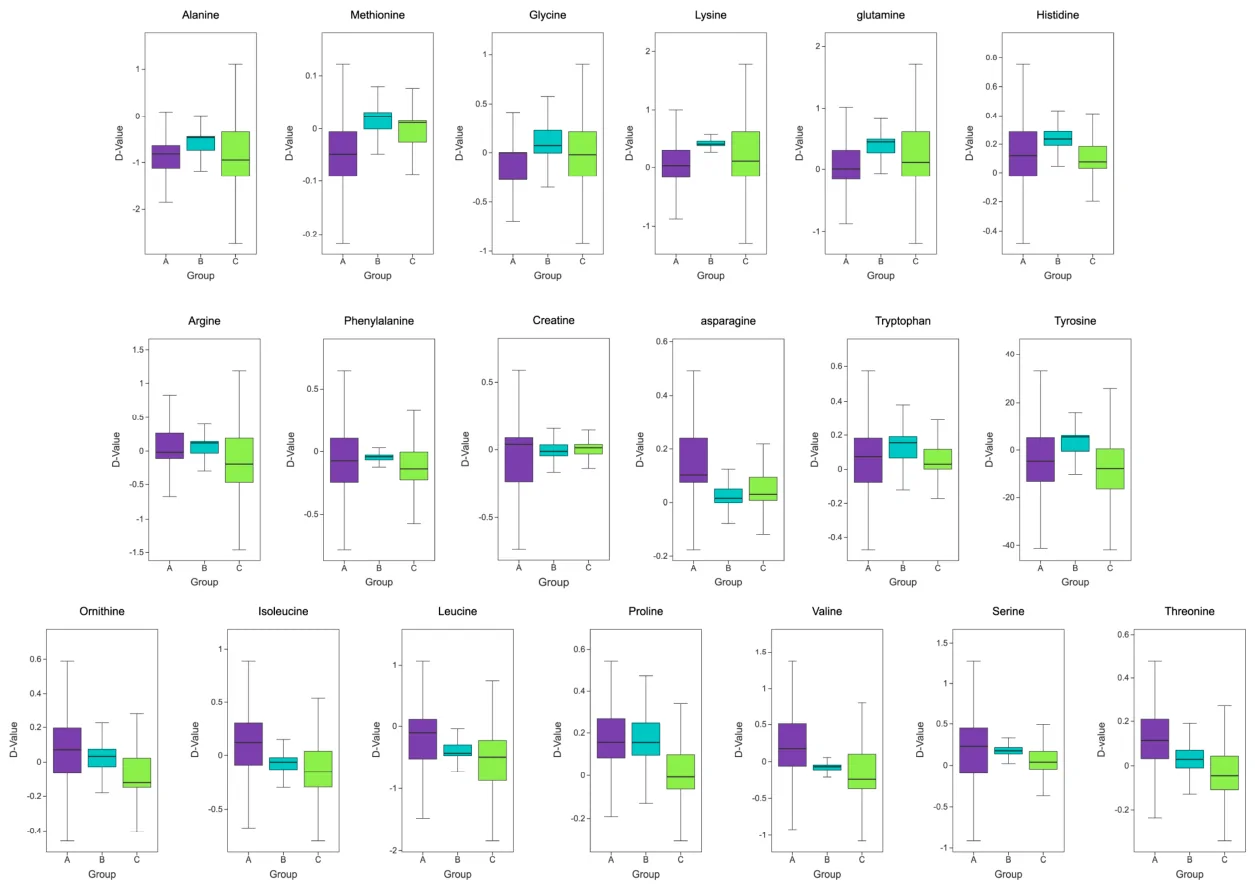
Boxplots of pre− and post−race plasma amino acid differences across three sweat stages. The y−axis represents the D−value (post−race minus pre−race difference) for each amino acid. Group A (purple), muddy sweat; Group B (teal), foamy sweat; Group C (green), clear sweat. Each panel corresponds to one of the 19 detected amino acids or derivatives.

The boxplot of pre- and post-race plasma amino acid differences showed that, overall, the B stage had the smallest dispersion in amino acid differences (Figure 8). Alanine, glycine, lysine, glutamine, and arginine showed smaller dispersion in A than in C, while serine, methionine, creatine, phenylalanine, histidine, ornithine, tryptophan, and asparagine showed larger dispersion in A than in C. Proline, leucine, valine, isoleucine, threonine, and tyrosine showed similar dispersion in A and C.

**Figure 9:**
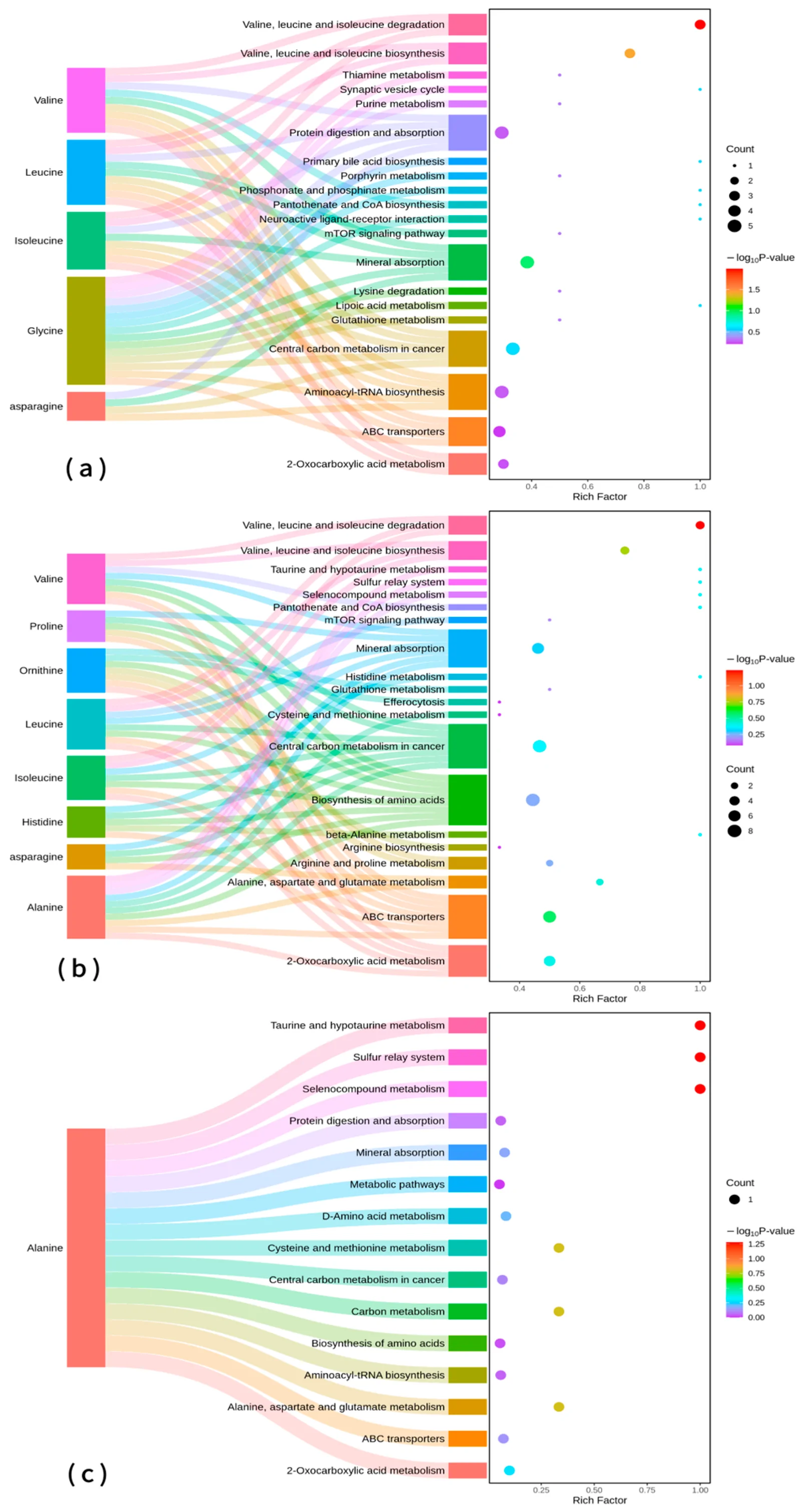
Sankey bubble plots of enriched pathways for differential plasma amino acids in the pre−race resting state. (**a**) A vs. B; (**b**) A vs. C; (**c**) B vs. C. **Left** panels show Sankey diagrams linking differential amino acids (**left**) to enriched KEGG pathways (**right**); **right** panels show bubble plots with rich factor on the x−axis, bubble size representing the count of enriched metabolites, and bubble color indicating −log_10_(*p*−value). Key shared pathways across comparisons include BCAA degradation, BCAA biosynthesis, mTOR signaling pathway, and ABC transporters.

Sankey bubble plots of differential amino acids in the pre-race resting state are shown in Figure 9. In A vs. B (Figure 9a), the main enriched pathways included BCAA degradation, BCAA biosynthesis, mTOR signaling pathway, ABC transporters, and mineral absorption, with differential amino acids valine, leucine, isoleucine, glycine, and asparagine being significantly enriched (*p* < 0.01). Glycine was additionally enriched in synaptic vesicle cycling, primary bile acid biosynthesis, and glutathione metabolism. In A vs. C (Figure 9b), BCAA degradation and mTOR signaling pathway were significantly enriched again; BCAA biosynthesis, taurine and hypotaurine metabolism, sulfur relay system, and ABC transporters also showed notable enrichment. In B vs. C (Figure 9c), only alanine was identified as a differential amino acid, which was significantly enriched (*p* < 0.01) enriched in taurine and hypotaurine metabolism, sulfur relay system, and ABC transporters.

**Figure 10:**
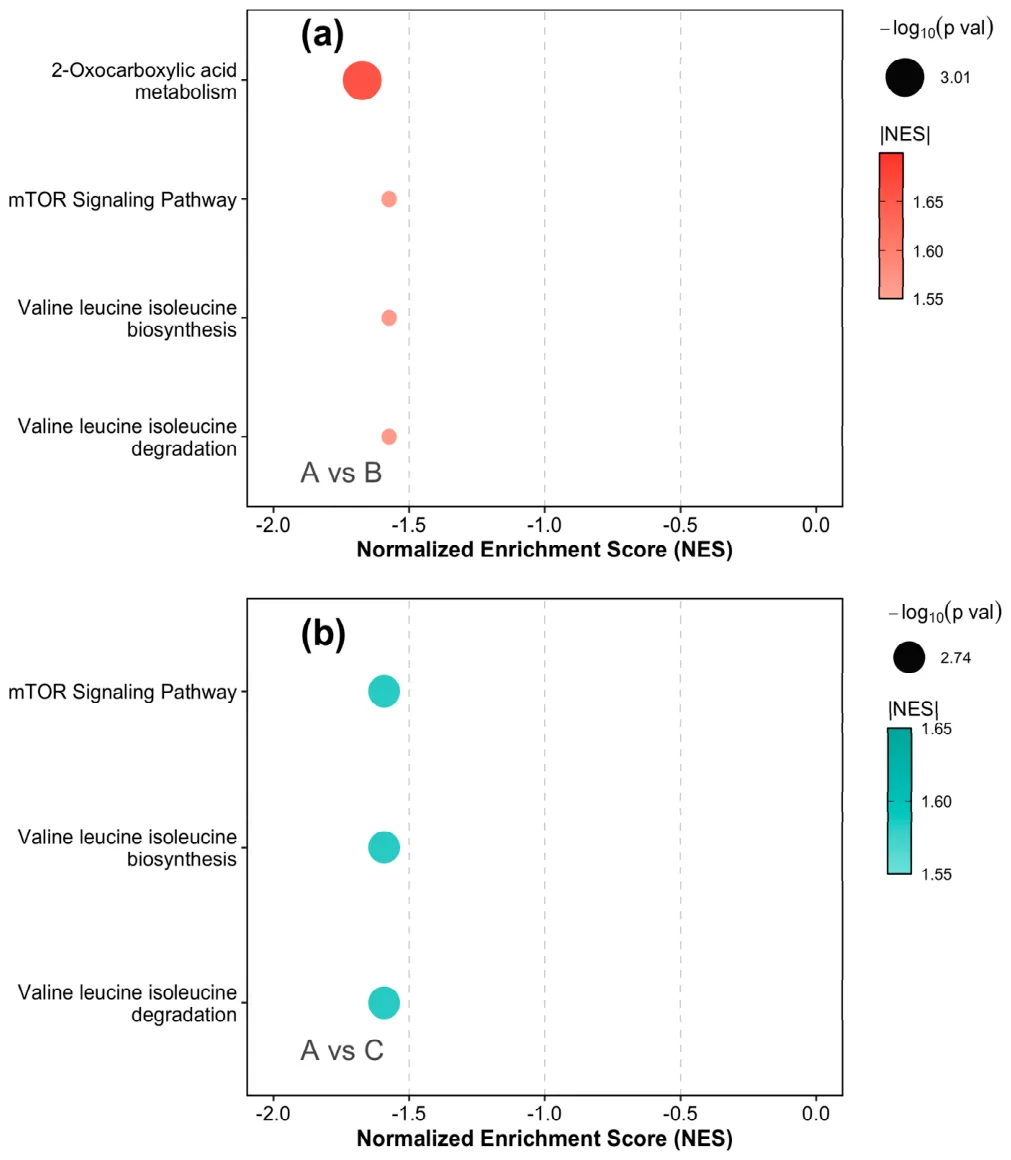
Metabolite set enrichment analysis (MSEA) of differential plasma amino acids between distinct sweat stages at rest. Bubble plots show significantly enriched pathways in (**a**) muddy sweat (MS) versus foamy sweat (FS, A vs. B) and (**b**) muddy sweat (MS) versus clear sweat (CS, A vs. C). The x−axis represents the normalized enrichment score (NES), bubble size corresponds to −log_10_(*p* value), and bubble color indicates the absolute value of NES (|NES|). All enriched pathways exhibited negative NES values, indicating a predominant downregulation of metabolites within these pathways in the MS stage.

To further quantify changes in plasma amino acid metabolic pathways, Metabolite set enrichment analysis (MSEA) was performed on the pre-race resting-state plasma amino acid differential profiles (Figure 10). In both A vs. B and A vs. C comparisons, BCAA degradation (NES ≈ −1.60, *p* < 0.01), BCAA biosynthesis (NES ≈ −1.60, *p* < 0.01), and mTOR signaling pathway (NES ≈ −1.60, *p* < 0.01) were significantly negatively enriched, with all NES values negative, indicating that these amino acid metabolic pathways were suppressed or undergoing remodeling in the MS stage relative to subsequent stages. The 2-oxocarboxylic acid metabolism pathway (NES ≈ −1.80, *p* < 0.01), unique to the A vs. B comparison, indicated multi-substrate energy mobilization in early conditioning. No significantly enriched pathways were detected in B vs. C, indicating that amino acid metabolic profiles between foamy and clear sweat stages had converged and metabolic homeostasis was established.

### 3.3. Correlation Patterns Between Sweat Differential Metabolites and Plasma Amino Acids

**Figure 11:**
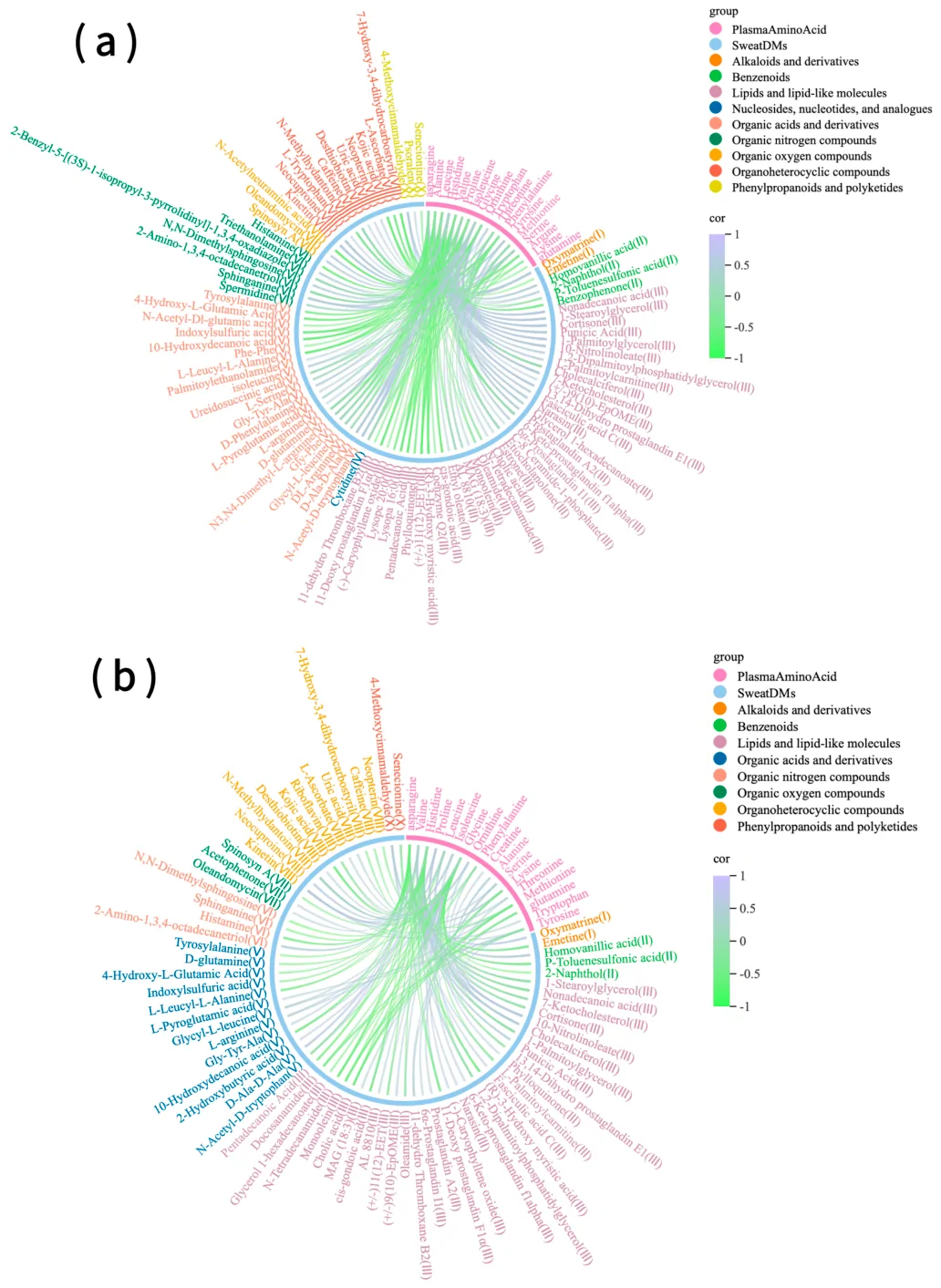
Chord diagrams illustrating correlations between sweat differential metabolites and plasma amino acids. (**a**) Post−race state; (**b**) pre−race resting state. Outer ring labels represent individual metabolites (pink, plasma amino acids; blue, sweat differential metabolites). Chord colors indicate correlation direction (purple, positive; green, negative). The thickness of each chord reflects the strength of correlation between paired metabolites.

To visualize the correlations between sweat differential metabolites and plasma amino acids, chord diagrams were constructed. In the post-race state (Figure 11a), plasma amino acids mainly correlated with organic acids and derivatives and lipids and lipid-like molecules among sweat differential metabolites, followed by organoheterocyclic compounds. Among 33 lipid differential metabolites, 19 showed negative correlations with plasma amino acids; among 22 organic acid differential metabolites, 7 showed negative correlations. Positive correlation chords were relatively sparse, revealing coordinated metabolic changes between systemic circulation and excretory systems immediately after exercise. In the pre-race resting state (Figure 11b), plasma amino acids mainly correlated with organic acids and derivatives, lipids and lipid-like molecules, and organoheterocyclic compounds among sweat differential metabolites. Among 39 lipid differential metabolites, 16 showed negative correlations with plasma amino acids; among 22 organic acid differential metabolites, 8 showed negative correlations. Positive correlation chords were sparser than in the post-race state, revealing that exercise training exerted coordinated metabolic effects on the excretory system and resting plasma amino acid reserves.

**Figure 12:**
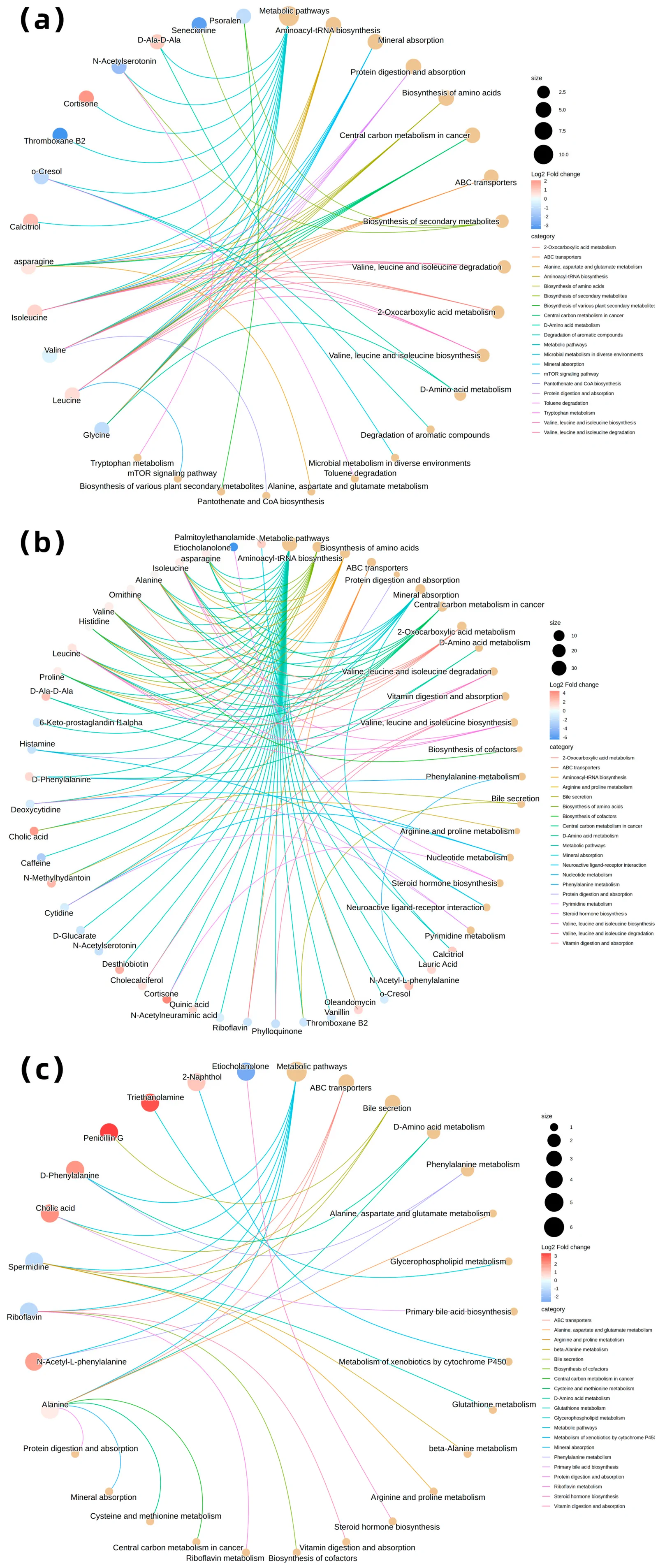
CNet network diagrams of co−enriched pathways between sweat metabolome and pre−race resting plasma amino acid metabolome. (**a**) A vs. B; (**b**) A vs. C; (**c**) B vs. C. Purple nodes represent enriched pathways; pink nodes represent plasma amino acids; blue nodes represent sweat differential metabolites. Edge thickness reflects the strength of enrichment association. Key shared pathways across comparisons include ABC transporters, mTOR signaling pathway, BCAA degradation, and BCAA biosynthesis.

CNet network diagrams of co-enriched pathways between sweat metabolome and pre-race resting plasma amino acid metabolome are shown in Figure 12. In A vs. B (Figure 12a), sweat differential metabolite D-Ala-D-Ala and plasma differential amino acid glycine formed an interconnected network through D-amino acid metabolism. BCAAs formed interconnected networks with cortisone and calcitriol through BCAA degradation and BCAA biosynthesis pathways. Aminoacyl-tRNA biosynthesis and amino acid biosynthesis pathways showed extensive connections with asparagine and glycine. The ABC transporter pathway was connected with four amino acids of different expression patterns. The mTOR signaling pathway was directly connected with leucine. In A vs. C (Figure 12b), sweat differential metabolite calcitriol formed an interconnected network with asparagine, BCAAs, alanine, and proline through mineral absorption. D-phenylalanine and D-Ala-D-Ala formed an interconnected network through D-amino acid metabolism. Palmitoylethanolamine and histidine formed a connection through neuroactive ligand–receptor interaction. Cholic acid and thromboxane B2 were connected with bile secretion. The ABC transporter pathway was connected with four differential metabolites of different expression patterns. Steroid biosynthesis was connected with etiocholanolone and cortisone. In B vs. C (Figure 12c), sweat differential metabolites spermidine and riboflavin formed an interconnected network with alanine through the ABC transporter pathway. Penicillin G, cholic acid, and spermidine were connected through bile secretion. These interconnected sweat differential metabolites and plasma differential amino acids demonstrate the metabolic remodeling of the circulatory-excretory system in *Yili* horses at different sweat stages during conditioning training adaptation.

## 4. Discussion

This study investigated the dynamic changes in sweat morphology during the conditioning of *Yili* horses and their association with systemic metabolic responses. For the first time, we employed integrated analysis of untargeted sweat metabolomics and targeted plasma amino acid metabolomics to reveal coordinated metabolic remodeling of the circulatory-excretory system under training stress. These findings not only provide a theoretical basis for assessing training adaptation status and optimizing training protocols in *Yili* horses but also lay the groundwork for future exploration of metabolic associations and regulatory networks in the circulatory-excretory system under exercise stress.

### 4.1. Sweat–Plasma Amino Acid Association Analysis

The morphological transition from A to B to C represents not only a visible manifestation of physiological adaptation to sustained exercise load but also reflects a profound shift from acute stress response in early training to stable adaptation in later stages. The MS stage represents acute stress, skin barrier remodeling, and cutaneous microbial homeostasis. At the initial stage of conditioning, *Yili* horses predominantly exhibited muddy sweat, with cortisone concentrations in MS horses showing significant upregulation relative to CS horses (FC = 22.44). Cortisone, a classic stress hormone [13], indicates that horses in early training are in a state of physiological stress and metabolic adaptation. Concurrently, thromboxane B2 (TXB2, FC = 0.10)—which is associated with inflammation and platelet activation—was lower in MS than in FS, suggesting that inflammatory stress response may be attenuated as the horse begins to adapt [14]. TXB2 is a stable metabolite of thromboxane A2 (TXA2), a key product of arachidonic acid metabolism with potent pro-platelet aggregation, vasoconstrictive, and pro-inflammatory properties [15,16,17]. In equine endotoxemia and shock models, TXA2 has been confirmed as an important pathophysiological mediator [18,19]. Exercise itself is a physiological stressor that induces mild inflammation; the downregulation of TXB2 in early training may thus signal a transition from initial stress toward adaptation.

Notably, 1-stearoylglycerol was significantly higher in MS than in FS (FC = 2.05). Monoacylglycerols are important components of skin lipids, and their upregulation in MS may represent a compensatory response to strengthen the skin barrier damaged by heavy sweating and physical friction [20,21]. The viscous nature of MS also implies low evaporative efficiency, associated with insufficient sweat gland activation in untrained horses [22,23]. In terms of pathway enrichment, vancomycin resistance and peptidoglycan biosynthesis—both bacterial-specific pathways—were significantly enriched at this stage. Sweat is not completely sterile; although the sampling area was cleaned prior to collection, sweat provides a unique environment for cutaneous microbiota, and microbial metabolites constitute part of the sweat metabolome [24,25]. While our study did not directly perform microbial sequencing, metabolomic data indicate that the skin microenvironment during the MS stage may differ markedly from that of subsequent stages. The rich presence of dander and dirt in MS provides a “breeding ground” for specific microorganisms. Peptidoglycan is a core structural component of bacterial cell walls and a target of many antibiotics including vancomycin [26]. D-Ala-D-Ala is an important component of peptidoglycan precursors [27], and its upregulation in MS corroborates the enrichment of peptidoglycan biosynthesis [28]. This suggests that the structure or metabolic activity of the cutaneous microbial community may undergo adaptive adjustments in response to changes in sweat physicochemical properties during early training.

The emergence of FS represents a landmark event in the training adaptation process. The foam in horse sweat is primarily caused by a surfactant protein called latherin [6,20,29], which significantly reduces water surface tension, facilitating sweat spreading across the hydrophobic hair coat and thereby increasing evaporative surface area and cooling efficiency [20,29]. During the MS-to-FS transition, dynamic changes in lipid and lipid-like molecules such as 1-stearoylglycerol (FC = 2.05) were observed. Stearic acid was also upregulated in MS vs. CS (FC = 1.98). These lipid molecules and their metabolites may influence foam formation by reducing sweat surface tension. Long-chain saturated fatty acids such as palmitic and stearic acid possess surfactant properties and can induce foam formation in anaerobic digestion [20]. We therefore hypothesize that as stearic acid concentrations rise, the excretion of these lipid molecules in sweat may act synergistically with latherin to promote foam formation and enhance heat dissipation. Anitaş also reported that palmitic acid concentrations in cow sweat can serve as a heat stress indicator, and high excretion of saturated and polyunsaturated fatty acids contributes to the formation of a water-lipid film on the skin surface that delays dehydration [30], providing corroborating evidence for the metabolic dynamics of fatty acids in *Yili* horse sweat and their influence on sweat physical properties. Furthermore, our previous study [31] found that sweat volume in *Yili* horses increased significantly with training. The emergence of the FS stage coincides with increased sweating volume and the demand for improved cooling efficiency. The foam formed through the synergistic action of latherin and fatty acids enables more uniform distribution of large sweat volumes across the body surface, avoiding the low evaporative efficiency of MS and thereby facilitating more effective heat loss. As a transitional stage between MS and CS, FS retains some stress characteristics of MS while beginning to exhibit adaptive changes seen in CS. The reduction in differential metabolite numbers from FS to CS indicates that the metabolic state of *Yili* horses is gradually stabilizing and their adaptation to training load is further improving.

The formation of CS marks precise physiological regulation and stable adaptation of the metabolic network to exercise training stress in *Yili* horses, consistent with the physiological observation that long-term training enhances athletic performance and endurance [22,32]. Compared with MS, CS showed significant downregulation of stress-related metabolites including cortisone and cholic acid, reflecting reduced stress levels and establishment of internal homeostasis. Primary bile acid biosynthesis and bile secretion pathways were significantly enriched in A vs. C, and multiple bile acids (e.g., cholic acid, FC = 13.99) were substantially higher in MS than in CS. This suggests that in early training, bile acids may reflect strong lipid mobilization and metabolic stress, whereas their return to stable concentrations in later training stages signifies adaptation of the energy regulatory system to exercise load [33,34]. Additionally, ureidosuccinic acid, an intermediate in de novo pyrimidine synthesis (FC = 0.04) [35], was continuously downregulated throughout the MS-to-CS transition, possibly related to training-induced adjustments in nucleic acid metabolism. The clear morphology and high evaporative efficiency of CS collectively reflect that after long-term training, energy supply becomes more efficient, inflammatory levels are effectively controlled, and homeostatic adaptation to exercise training load is achieved.

In summary, the transition of sweat morphology in *Yili* horses is a complex, multi-layered biological process involving coordinated regulation of stress response, lipid metabolism, cutaneous microenvironment, and surfactant substances. This dynamic process clearly delineates the metabolic remodeling trajectory from initial training to adapted status.

### 4.2. Differences in Plasma Amino Acids Before and After the Test Race Across Distinct Sweat Morphologies

For clarity, Groups A-C refer to the three sweat stages obtained from the same six horses at progressive time points during the 10-week conditioning program: Group A corresponds to muddy sweat (MS, approximately weeks 1–3), Group B to foamy sweat (FS, approximately weeks 4–6), and Group C to clear sweat (CS, approximately weeks 7–10).

Through targeted metabolomics, this study systematically revealed, for the first time, the plasma amino acid metabolic characteristics associated with three distinct sweat phenotypes—muddy sweat, foamy sweat, and clear sweat—during the conditioning of *Yili* horses. Although these terms are used in equine training practice to describe sweat appearance, they have not been clearly defined or biochemically classified in the scientific literature [36,37]. These distinct sweat phenotypes are closely associated with unique plasma amino acid profiles and metabolic pathway activities, reflecting physiological stress and adaptation at different training stages.

The conditioning program followed a progressive training protocol where the intensity and duration of exercise were gradually increased over the 10-week period (Table S1). Horses were exposed to a structured exercise regimen including round-pen and track training, with the 2000 m test race serving as a standardized physiological challenge at the end of each training phase.

In the pre-race resting state, the MS group exhibited a metabolic signature significantly associated with branched-chain amino acid (BCAA) metabolism. Compared with groups B and C, group A showed significantly higher plasma BCAA concentrations (*p* < 0.05). BCAAs are essential amino acids that serve not only as important substrates for protein synthesis in skeletal muscle but also as energy substrates and signaling molecules regulating protein metabolism [38]. Studies have shown that horses consume BCAAs during endurance exercise, leading to decreased plasma concentrations [11]. Therefore, the elevated resting BCAA concentrations in the MS group may reflect higher rates of muscle protein catabolism in early training, resulting in BCAA release into the bloodstream, or alternatively, downregulation of BCAA oxidative catabolism leading to their accumulation in blood [39,40]. Pathway enrichment results support both interpretations: BCAA degradation and BCAA biosynthesis pathways were significantly enriched in comparisons between MS and the other two groups, indicating that the entire BCAA metabolic network in MS is in a highly active or remodeled state to meet the metabolic demands of early training. Concurrently, glycine (Gly) was significantly downregulated in MS (*p* < 0.05), which may be related to increased demand for collagen, creatine, and glutathione—substances critical for tissue repair and antioxidant defense. Glycine is a major component of collagen, and its consumption may reflect ongoing connective tissue microdamage or repair needs during the MS stage [39]. Glutathione (GSH) is a core antioxidant molecule whose synthesis depends on glycine [41]. Gly downregulation may reflect that MS horses face higher oxidative stress and require substantial GSH synthesis to clear reactive oxygen species (ROS) generated by exercise. The differences between FS and CS at rest were relatively minor, with FS showing higher alanine (Ala) concentrations (*p* < 0.05). Alanine is the core carrier of the glucose–alanine cycle, responsible for transporting muscle amino nitrogen to the liver for glucose synthesis. The higher Ala concentrations in FS suggest that even at rest, an active gluconeogenic process exists, possibly pre-stocking energy for exercise. This process was confirmed by Miller et al. [42], who demonstrated that young horses rely on protein and amino acid catabolism to maintain blood glucose after exercise. The significant enrichment of “protein digestion and absorption” in our pathway analysis also supports this view.

The 2000 m high-intensity test race, as a strong physiological stressor, further amplified metabolic differences between sweat phenotypes. After exercise, the MS group showed persistently elevated plasma valine (Val) compared with the other two groups, while histidine (His) and aspartate (Asp) were also significantly upregulated. The persistent elevation of valine again points to BCAA metabolism as the core amino acid metabolic feature distinguishing the MS phenotype. Histidine is a precursor for carnosine synthesis; carnosine possesses powerful pH buffering and antioxidant capacity in muscle [43]. Post-exercise histidine elevation may reflect enhanced defense mechanisms in MS horses to counteract acidosis and oxidative stress induced by strenuous exercise. Aspartate directly participates in the tricarboxylic acid cycle and urea cycle; its elevation indicates strengthened mobilization of amino acids as energy substrates and enhanced processing of metabolic byproducts. Pathway enrichment analysis showed that post-exercise aminoacyl-tRNA biosynthesis, amino acid biosynthesis, and protein digestion and absorption pathways were activated in stage A, indicating accelerated protein turnover, repair, and synthesis after exercise. This further demonstrates that the appearance of muddy sweat during conditioning represents a stress-regulatory response of the organism to exercise load.

The FS and CS groups showed no statistically significant differences in post-race plasma amino acids. Although subtle differences existed at rest, their amino acid metabolic responses converged when facing high-intensity, high-load test races. Under constant feeding conditions, the sequential transition from A→B→C in sweat morphology reflects progressive depletion of amino acid reserves as the training program advances and training load intensifies. The MS group showed significant upregulation of Val, Leu, and Ile at rest, whereas the CS group showed only slight upregulation of Ala, indicating decreasing BCAA reserves from A to C. After exercise, group A maintained Val upregulation, but stage C showed no differential amino acids. Long-term training enhances fatty acid oxidation capacity [9,44,45], while ABC transporter-mediated metabolic clearance systems are upregulated to ensure efficient excretion of nitrogenous wastes and maintenance of plasma amino acid dynamic balance. This marks a qualitative shift in metabolic regulation from acute stress response to efficient homeostatic maintenance.

The KEGG annotation rate for sweat metabolites was approximately 38%, which reflects the inherent challenge of annotating complex biological matrices such as equine sweat. Sweat contains a high proportion of lipids, lipid-like molecules, and uncharacterized small molecules that are not well-represented in current metabolite databases. This annotation rate is comparable to reports from other sweat metabolomics studies in humans and livestock. While this limitation may affect the comprehensiveness of pathway enrichment analysis, the core findings regarding amino acid metabolism and energy pathways remain robust, as these well-annotated metabolite classes were consistently detected across all samples.

### 4.3. Plasma–Sweat Metabolic Association and Pathway Co-Regulation

The preceding analyses have separately elucidated the differential metabolite profiles during sweat morphology transitions and the dynamic changes in plasma amino acid concentrations accompanying this process. This section explores the intrinsic connections between the two, elaborating on the coordinated actions between plasma amino acid mobilization, consumption, and sweat metabolite excretion during high-intensity exercise from three perspectives: metabolite correlations, transmembrane transport mechanisms, and core signaling pathways.

To macroscopically understand the relationship between plasma amino acids and sweat metabolites, we first analyzed their overall correlation patterns under different physiological states (Figure 11). At the pre-exercise resting state, plasma amino acids showed broad positive correlations with lipids and lipid-like molecules and organic acids and derivatives in sweat (Figure 11b). This indicates that under basal metabolic conditions, plasma and sweat components maintain a relatively synchronous balance; as a product of plasma filtration and glandular secretion, sweat metabolite composition reflects the basal metabolic level of the organism.

However, after the high-intensity test race, this correlation reversed. Post-race plasma amino acid concentrations showed significant negative correlations with organic acids and derivatives and lipids and lipid-like molecules in sweat (Figure 11a). This shift clearly reveals exercise-induced metabolic remodeling. During exercise, to meet enormous energy demands and cope with oxidative stress, the organism mobilizes substantial lipids and plasma amino acids—especially BCAAs [43,46]—generating degradation products of lipids and organic acids. These products increase in concentration in tissues and systemic circulation and are excreted in large quantities through sweat to maintain internal environment stability [47]. Thus, the abundance of consumed amino acids in plasma decreases while that of organic acids and lipid molecules as metabolic wastes in sweat increases, forming a strong negative correlation pattern. This indicates that post-exercise sweat excretion serves not only as the primary means of thermoregulation but also as an important pathway for excreting exercise metabolic byproducts and reflecting metabolic intensity and stress [48].

Building upon overall correlations, we further identified that specific correlations between plasma amino acids and sweat metabolites revealed several potentially critical metabolic regulatory axes. Analysis showed that in both resting (Figure 11b) and post-exercise (Figure 11a) states, plasma leucine and valine concentrations were positively correlated with glycerol esters such as 1-palmitoylglycerol and 1-stearoylglycerol in sweat. BCAAs are important energy sources for muscle tissue; during prolonged or high-intensity exercise, their oxidative decomposition provides ATP [49,50,51]. Concurrently, the organism decomposes triglycerides through lipolysis, producing glycerol and fatty acids; glycerol esters as metabolic intermediates also increase. The positive correlation between plasma BCAAs and sweat glycerol esters indicates that during high-intensity exercise, protein catabolism marked by BCAA consumption and lipid catabolism marked by glycerol ester excretion are two parallel-activated, synergistic core energy supply pathways.

In contrast to BCAAs, plasma glycine and serine showed negative correlations with alkaloids and uric acid in sweat (Figure 11a,b). Glycine and serine are precursors for purine synthesis, while uric acid is the end product of purine metabolism and is often regarded as a marker of cell turnover and oxidative stress. Alkaloid substances are typically endogenous or exogenous nitrogenous metabolic wastes. This negative correlation may indicate that under high-intensity exercise stress, the organism may prioritize utilizing glycine and serine for more critical physiological processes such as glutathione synthesis to combat oxidative stress, leading to reduced utilization in plasma; meanwhile, various nitrogenous wastes produced due to enhanced metabolism and tissue decomposition are accelerated for clearance through sweat.

A third regulatory axis involves the proline–uric acid positive correlation and the creatine–prostaglandin negative correlation. The positive correlation between proline and sweat uric acid (Figure 11b) may reflect that both are associated with high-energy operation of the organism. Creatine, as the core of the phosphocreatine energy system, showed a negative correlation in plasma with prostaglandin E1 in sweat—a signal molecule reflecting inflammation and cellular stress (Figure 11b)—possibly revealing the organism’s transition from anaerobic instantaneous energy supply systems to stress-inflammatory response states.

Through pathway co-enrichment and CNet analysis, this study identified three key co-regulatory pathways, among which the ABC transporter pathway was a core finding. During the A-to-B sweat transition in *Yili* horses, this pathway was co-enriched by both plasma differential amino acids and sweat differential metabolites. CNet network diagrams further showed that the ABC transporter pathway is connected with multiple differentially expressed amino acids (Figure 12a,c). ABC transporters are a family of ATP-dependent proteins mediating bidirectional transmembrane transport of amino acids, lipids, and metabolites; however, their molecular mechanisms for regulating specific substrate transport in skin and sweat glands remain to be elucidated [52,53,54,55,56,57]. The concentration of amino acids in sweat can sometimes far exceed that in plasma [58,59], strongly implying the existence of active transport mechanisms. Multi-omics association analysis results indicate that amino acids, lipids, and organic acids and other small molecules may be actively excreted from the circulatory system into sweat through sweat glands mediated by ABC transporters. Although functional validation at the transporter level requires further experimental confirmation, this study has revealed the potential key role of ABC transporters in sweat excretion mechanisms, laying a foundation for subsequent in-depth exploration of their regulatory networks in exercise adaptation.

The second key pathway involves the coordinated regulation of BCAA metabolism and mTOR signaling. In the BCAA metabolic pathway, plasma BCAAs not only underwent significant changes themselves but were also closely linked to BCAA biosynthesis and BCAA degradation pathways. During the high-load exercise training phase corresponding to the MS-to-FS sweat transition in *Yili* horses, upregulated leucine and isoleucine were significantly enriched in BCAA-related pathways (Figure 10a). This again demonstrates that BCAA catabolism is a key energy metabolic pathway for *Yili* horses responding to high-intensity exercise stress, and the sweat morphology transition from MS to CS corresponds to a period of highly activated BCAA metabolism in vivo. Simultaneously, the BCAA-mTOR signaling pathway enriched in plasma amino acids represents a breakthrough finding of this study; CNet networks showed that plasma leucine is directly connected with the mTOR signaling pathway (Figure 10a). mTOR (mammalian target of rapamycin) is a central regulatory hub for cell growth, proliferation, and metabolism, and leucine is one of the most important signal molecules activating the mTORC1 complex [56,60,61,62]. During exercise, muscle protein breakdown leads to fluctuations in plasma leucine concentrations; this change is not merely energy substrate consumption but a strong cellular signal. By activating the mTOR pathway, changes in leucine can influence downstream protein synthesis, autophagy, and glucose-lipid metabolism, thereby achieving macroscopic regulation of the organism’s overall metabolic state [63]. The plasma-sweat metabolome association analysis revealed the phenomenon of amino acids as signaling molecules initiating key cellular regulatory networks, while also revealing their loss as energy substances in *Yili* horses during training. These results emphasize that sweat excretion is not simply water loss but a key physiological process for precisely regulating material balance and energy utilization.

### 4.4. Study Limitations and Future Perspectives

It is important to acknowledge that this study was based on a relatively small cohort (*n* = 6). While the longitudinal within-subject design partially mitigates the impact of limited sample size by using each horse as its own control, the statistical power for detecting subtle metabolic differences may be reduced. Multivariate analyses (PCA, PLS-DA, OPLS-DA) are generally robust to moderate sample sizes, but the generalizability of our findings to larger populations should be interpreted with caution. Future studies with expanded cohorts (n ≥ 15–20) are warranted to validate the metabolic signatures identified here.

The involvement of ABC transporters is inferred from pathway enrichment analysis and co-expression patterns. While our data suggest a potential role for these transporters in facilitating amino acid flux between plasma and sweat compartments, direct functional evidence (e.g., pharmacological inhibition or transporter-specific assays) is required to establish causality.

In terms of practical application, this study proposes that plasma BCAA concentrations may serve as potential molecular markers of training stress intensity, sweat cortisol and plasma BCAA as potential candidate biomarkers of training stress that warrant validation in larger, independent cohorts, while sweat cortisol may be used as a non-invasive monitoring indicator for equine welfare assessment. Training intensity should be adjusted according to sweat morphology: appropriately reduced during the MS stage, maintained during the FS stage, and gradually increased during the CS stage. We acknowledge that muddy sweat (MS) samples may contain higher levels of skin-derived contaminants (e.g., keratin, sebum, environmental debris) compared to foamy and clear sweat samples. The elevated concentrations of certain lipids and organic acids in MS may partly reflect this contamination rather than purely exercise-induced metabolic changes. However, the consistent patterns observed across all six horses and the gradual metabolic transition from MS to CS suggest that biological adaptation signals predominate over contaminant effects. Future studies employing controlled skin cleaning protocols and contaminant subtraction methods could further clarify this issue.

## 5. Conclusions

The sequential transition from muddy sweat (MS) to foamy sweat (FS) to clear sweat (CS) during the conditioning of *Yili* horses corresponds to three physiological stages: acute stress, transitional adaptation, and homeostatic maintenance, with sweat cortisol serving as a non-invasive indicator of training stress. The MS stage is characterized by significant changes in lipid metabolites (e.g., 1-stearoylglycerol) and stress hormones in sweat, while the FS and CS stages exhibit convergent sweat metabolome profiles. Correspondingly, the MS stage shows significantly elevated plasma branched-chain amino acid (BCAA) concentrations and decreased glycine, with BCAA metabolism representing the core amino acid feature distinguishing the MS phenotype. Integrative analysis revealed that ABC transporters, the mTOR signaling pathway, and BCAA metabolic pathways play key roles in plasma–sweat co-regulation, forming two core metabolic regulatory axes: the BCAAs–glycerol ester energy mobilization axis and the glycine/serine–alkaloid/uric acid metabolic clearance axis. These findings indicate that transmembrane transport of sweat metabolites under exercise stress is actively regulated by ABC transporters rather than passive diffusion. The above findings provide a theoretical basis for assessing training adaptation status and optimizing training protocols in *Yili* horses.

## Figures and Tables

**Table 1:** Time of sweat morphology change.

Sweat Morphology	Time(d)				
	Minimum (d)	Maximum (d)	Average		
			MS→FS (Mean ± SD) (d)	FS→CS (Mean ± SD) (d)	MS→CS (Mean ± SD) (d)
Muddy sweat	0	0	20.33 ± 2.34		55 ± 2.97
Foamy sweat	18	24		34.67 ± 2.16	
Clear sweat	50	59			

## Data Availability

The raw metabolomics data reported in this paper have been deposited in the OMIX, China National Center for Bioinformation/Beijing Institute of Genomics, Chinese Academy of Sciences (https://ngdc.cncb.ac.cn/omix) (accessed on 31 May 2026) under accession number OMIX017327.

## Supplementary Materials

The following supporting information can be downloaded at: https://www.mdpi.com/article/10.3390/biology15131033/s1, Table S1: Breaking and conditioning program for *Yili* horses during the 10-week conditioning period.

## Abbreviations

The following abbreviations are used in this manuscript:

MS	muddy sweat
FS	foamy sweat
CS	clear sweat
BCAA	branched-chain amino acid
PCA	principal component analysis
PLS-DA	partial least squares discriminant analysis
OPLS-DA	orthogonal partial least squares discriminant analysis
VIP	variable importance in projection
FC	fold change
MSEA	metabolite set enrichment analysis
KEGG	Kyoto Encyclopedia of Genes and Genomes
CNet	co-enrichment network
LC-MS/MS	liquid chromatography-tandem mass spectrometry
UHPLC-MS/MS	ultra-high performance liquid chromatography-tandem mass spectrometry
MRM	multiple reaction monitoring
NES	normalized enrichment score
