# Correction: Xue et al. Untargeted Sweat Metabolomics and Targeted Plasma Amino Acid Profiling Reveal Dynamic Metabolic Remodeling During Conditioning in Yili Horses. *Biology* 2026, *15*, 1033

**DOI:** 10.3390/biology15171565

**Published:** 2026-09-07

**Authors:** Yuheng Xue, Penghui Luo, Zhehong Shen, Chen Meng, Xinkui Yao, Jun Meng, Wanlu Ren, Tongliang Wang, Yaqi Zeng

**Affiliations:** 1College of Animal Science, Xinjiang Agricultural University, Urumqi 830052, China; xyh9523@126.com (Y.X.); 18858657690@163.com (Z.S.); chenmeng0330@126.com (C.M.); yaoxinkui@xjau.edu.cn (X.Y.); junm86@xjau.edu.cn (J.M.); renwanlu@xjau.edu.cn (W.R.); wtl13639911402@163.com (T.W.); 2Xinjiang Key Laboratory of Horse Breeding and Exercise Physiology, Urumqi 830052, China; 3Horse Industry Research Institute, Xinjiang Agricultural University, Urumqi 830052, China; 4Xinjiang Uygur Autonomous Region Animal Husbandry General Station, Urumqi 830052, China; xmzztxzx1@163.com

## References

In the original publication [1], we found errors in References [19] and [45], caused by a DOI auto-matching malfunction in our reference management software. During bibliography generation, the software incorrectly assigned DOIs to these references, resulting in mismatches between the cited articles and their DOI links. The order of the references has not been changed. The corrected references appear below. The authors state that the scientific conclusions are unaffected. This correction was approved by the Academic Editor. The original publication has also been updated.

Ref [19]: Carmona, R.H.; Tsao, T.C.; Trunkey, D.D. The role of prostacyclin and thromboxane in sepsis and septic shock. *Arch. Surg.* **1984**, *119*, 189–192. https://doi.org/10.1001/archsurg.1984.01390140053009.

Ref [45]: Kitaoka, Y.; Mukai, K.; Aida, H.; Hiraga, A.; Masuda, H.; Takemasa, T.; Hatta, H. Effects of high-intensity training on lipid metabolism in Thoroughbreds. *Am. J. Vet. Res.* **2012**, *73*, 1813–1818. https://doi.org/10.2460/ajvr.73.11.1813.
