# Supplementary material for: Untargeted Sweat Metabolomics and Targeted Plasma Amino Acid Profiling Reveal Dynamic Metabolic Remodeling During Conditioning in Yili Horses
Source: Biology (Basel). 2026 Jun 28;15(13):1033. doi: 10.3390/biology15131033 (PMC13359863; doi:10.3390/biology15131033)
Supplement: Supplementary file 1 [file biology-15-01033-s001.zip › biology-4365063-supplementary.pdf]

Table S1 Breaking and Conditioning program

Stages	Time/ week	Conditioning Program
Stage 1	1	Round-pen training 1.5 h/d, completing horse gentling and saddling training; completing mounting training, accepting the rider, and responding to simple commands
Stage 2	2	Round-pen lunging or hot-walker warm-up 45 min, round-pen riding 15 min, training on both left and right rein
Stage 3	3	Round-pen lunging or hot-walker warm-up 40 min, riding 20 min; arena riding training: 6 horses per group, performing serpentine, figure-eight, "Z", circle, and other patterns in a fixed arena under the lead of a lead horse; improving horse flexibility, coordination, and obedience, while gradually relaxing the horse through training and acclimating it to extended riding periods
Stage 4	4	Round-pen lunging or hot-walker warm-up 35 min, riding 25 min; track trotting training: horses enter the track, walk 800 m, then trot 800 m, then walk 800 m before returning to the stable
	5	Round-pen lunging or hot-walker warm-up 35 min, riding 25 min; track trotting training: horses enter the track, walk 800 m, then trot 1600 m, then walk 800 m, with horses crossing each other during the walking phase, before returning to the stable
Stage 5	6	Round-pen lunging or hot-walker warm-up 30 min, riding 30 min; track riding training: horses enter the track in groups under the lead of a lead horse. Walk 800 m, trot 800 m, canter 600 m, walk 1000 m
	7	Round-pen lunging or hot-walker warm-up 30 min, riding 30 min; track riding training: horses enter the track in groups under the lead of a lead horse. Walk 800 m, trot 800 m, canter 1200 m, walk 1200 m
Stage 6	8	Round-pen lunging or hot-walker warm-up 30 min, riding 30 min; track trotting warm-up: horses enter the track in groups, walk 800 m and trot 800 m under the lead of a lead horse to complete warm-up, then proceed to the grass training field: horses enter a fenced grass field with landmarks for training. Horses complete a 2400 m one-way straight canter in a designated direction. After three days of grass training, the fixed lead horse can be omitted, with each horse alternating as the lead horse to build confidence
	9	Round-pen lunging or hot-walker warm-up 30 min, riding 30 min; track trotting warm-up: horses enter the track in groups, walk 800 m and trot 800 m under the lead of a lead horse to complete warm-up, then proceed to the grass training field: horses enter a fenced grass field with landmarks for training. Horses complete a 2400 m straight canter (1200 m out and back). After three days of grass training, the fixed lead horse can be omitted, with each horse alternating as the lead horse to build confidence
Stage 7	10	Round-pen lunging or hot-walker warm-up 30 min, riding 30 min; track trotting warm-up: horses enter the track in groups, walk 800 m and trot 800 m under the lead of a lead horse to complete warm-up, then proceed to the training field for gallop transition training: gradually increasing speed in the track until the horse's gait transitions to gallop, but without whipping or pushing the horse, until the horse naturally transitions from canter to gallop
